# Biennial sub-meter tree coverage dataset of Orlando (2013–2021)

**DOI:** 10.1038/s41597-025-05059-9

**Published:** 2025-04-29

**Authors:** Danlu Cai, Leiqiu Hu, Janak Parajuli

**Affiliations:** Department of Atmospheric and Earth Science, University of Alabama in Huntsville, Huntsville, AL 35899 USA

**Keywords:** Sustainability, Climate-change mitigation

## Abstract

Rapid urban development often comes at the cost of significant vegetation loss, and the loss of urban trees is a particularly concerning issue. Many cities have recognized the crucial role of tree canopy cover in addressing a range of pressing environmental challenges, and have set goals in their development plans. However, the lack of resources for monitoring its spatiotemporal changes directly undermines efforts of cities to balance urban growth with environmental sustainability. We developed high-resolution biennial tree canopy maps (2013–2021) for the Orlando metropolitan area using National Agricultural Imagery Program (NAIP) orthoimagery. Our approach addresses challenges such as sensor inconsistencies, shadows, variable observation times, and environmental factors. Extensive ground-truth validation demonstrates 78%–89% accuracy in tree detection, outperforming existing products. This dataset provides the crucial link between planning and action, and supports urban forest management and has broader applications in urban planning, environmental and public health monitoring, and education programs.

## Background & Summary

Trees provide critical ecological, societal, psychological, and physical benefits in urban systems. Besides their aesthetic functions, planting and maintaining urban trees are one of the most widely adopted nature-based solutions to address complex urban environmental and climate challenges simultaneously over the world^1–6^. For example, it is widely acknowledged the cooling benefits of urban trees that mitigate the urban heat island effects^7–10^ with reduced energy consumption for cooling^11,12^, mitigate some of the health effects from air and runoff pollution by absorbing harmful pollutants from the air^13,14^ and by intercepting and filtering stormwater runoff^15,16^. Urban forests also support fauna and flora within the highly compact human habitats, which preserve and promote biodiversity in delicate urban ecosystems^17–19^. Thus, urban trees and/or urban greenery have been increasingly recognized as a crucial component of achieving the United Nations Sustainable Development Goal (SDG) 11 in creating cities and human settlements more sustainable, safe, resilient, and inclusive^20,21^.

Urban environments are highly managed by human activities and constantly experience changes referring not only to variation in the physical environment over time, but also associated with disturbances from climate change and extreme events^22–26^. Consequently, urban trees tend to change more significantly over time compared to other human-made structures like buildings and roads. Partly, the governance of urban green spaces is influenced by management decisions at various levels, including institutions, municipalities, and individual landowners. A form of artificial rather than natural selection occurs, showing substantial disparities in the tree diversity, composition, structure and spatial distribution over multiple green space types in the public and private domain^27^. In addition, individual tree species respond differently to environmental stressors and management conditions due to their varying environmental requirements and species sensitivity and adaptivity^28^. Routinely tracking tree canopy distribution and health conditions is essential for effective management of urban forests, enabling cities to maximize the environmental benefits, such as air purification, temperature regulation, and carbon sequestration, while also identifying potential threats to tree health like pests, diseases, or climate extremes^29,30^.

The Orlando metropolitan area, among the fastest-growing U.S. regions^31^, has faced accelerated tree loss driven by frequent extreme weather events, including hurricanes and tropical storms^32–35^. In its 2013 Community Action Plan, the City of Orlando—a major developed municipality—set a goal to increase tree canopy coverage from ~23% to 40% by 2040. Municipal authorities, local organizations, and individuals have implemented initiatives such as the One Person, One Tree policy^36^ to replant trees and restore diminished canopy coverage. However, since the plan’s adoption, no systematic resources have been available to monitor canopy changes over time—a critical gap for evaluating progress, informing adaptive management, and ensuring alignment with the 2040 target.

Many existing tree canopy datasets suffer from coarse spatial resolutions and infrequent updates, limiting their ability to capture dynamic urban tree canopy changes. For example, the US Department of Agriculture (USDA) Forest Service’s Tree Canopy Cover (TCC) maps-- part of the National Land Cover Database (NLCD) --provide 30-meter resolution estimates updated every five years (2001–2021)^37^. While valuable for national-scale trends, this resolution is inadequate for urban landscapes, where complex mixtures of impervious surfaces and vegetation create fine-scale heterogeneity. Automated global products like Global Forest Watch^38,39^ (10 m Sentinel-2-based) and Google’s Dynamic World^40^ (10 m resolution) similarly struggle to resolve urban tree canopies due to pixel-level ambiguities. The USDA Forest Service i-Tree^41^ offer tools for estimating canopy coverage percentages within user-defined boundaries through manual interpretation of aerial imagery^42,43^. However, the output lack spatial explicit maps, and accuracy depends heavily on operator’s expertise and sample size^42,44,45^. While commercial platforms (e.g., Planet Labs’ 3–5 m imagery, Airbus OneAtlas’ 0.5 m data, and PlanIT Geo) provide higher resolution, their costs are often prohibitive for municipalities managing long-term monitoring programs.

To address these gaps, our product delivers high-resolution, biennial, and cost-effective tree canopy maps tailored to urban complexity—a critical advancement for cities like Orlando pursuing data-driven tree canopy restoration. Using orthorectified multispectral aerial imagery (2013–2021), we developed and validated a replicable methodology to map tree canopy dynamics across the Orlando metropolitan area. Our approach, detailed in this study, enables scalable application to other cities or future updates. The resulting dataset provides unprecedented spatial and temporal resolution, capturing fine-scale canopy distribution patterns essential for targeted urban forestry management. Beyond supporting policy decisions (e.g., prioritizing tree planting in vulnerable neighborhoods), this resource empowers interdisciplinary research in urban climate, ecology, environmental justice, and socio-economic studies. By democratizing access to actionable canopy data, our work bridges the gap between municipal planning needs and scientific innovation.

## Methods

### Aerial imagery

Our tree canopy maps were derived from 1-meter to 0.6-meter resolution aerial imagery acquired by the National Agriculture Imagery Program (NAIP), which conducts biennial surveys across the continental United States (available at: https://earthexplorer.usgs.gov/, and boundary available at: https://www.census.gov/cgi-bin/geo/shapefiles/index.php?year=2020&layergroup=Urban+Areas). NAIP’s spatial resolution has been improved from 1-meter (2003–2017) to 0.6-meter post-2018, enhancing fine-scale urban feature detection. The dataset includes four spectral bands: red, green, blue, and near-infrared (NIR), with the NIR band significantly enhancing vegetation discrimination. To mitigate shadow effects^46^, NAIP flights follow a north-south flight path during periods of high solar elevation and were timed to coincide with peak growing seasons to maximize vegetation visibility. All imagery is freely accessible via the USGS EarthExplorer. For detailed metadata on acquisition protocols, radiometric calibration, and positional accuracy, refer to the NAIP Program Description^47^.

Between 2013 and 2021, NAIP imagery acquisition in Florida utilized multiple sensor models and serial numbers, introducing variability in spectral band specifications. For examples, imagery was collected using ADS40-SH51 and ADS40-SH91 sensors in 2013, ADS-80SH81 in 2015, and ADS-100 in 2015–2021. These sensors differ in spectral band wavelength ranges. For instance, the ADS-100 captures red (619–651 nm), green (525–585 nm), blue (435–495 nm), and NIR (808–882 nm), while the ADS-80 records red (604–664 nm), green (533–587 nm), blue (420–492 nm), and NIR (833–920 nm). Such variations cause inconsistencies in spectral and radiometric responses, altering histogram distributions, spectral combinations, and vegetation index calculations for identical land cover features. Additionally, solar angles vary in each survey, as do atmospheric and vegetation conditions. To ensure comparability across years, we developed methods that harmonize these sensor-derived discrepancies in the source data.

### Urban tree canopy mapping framework

**Fig. 1:**
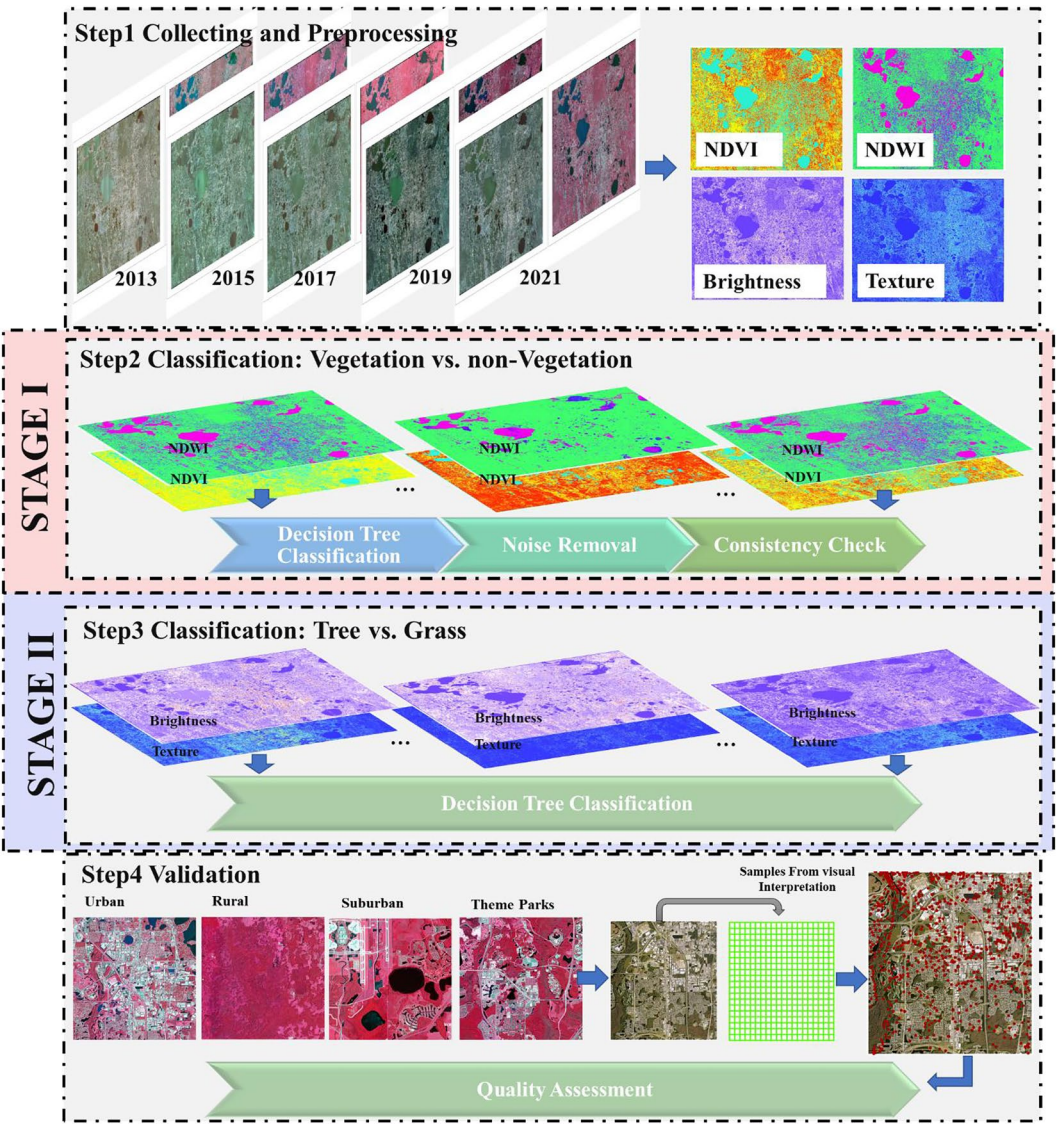
Framework for tree canopy detections from ariel images over the Orlando metropolitan area.

We developed a hierarchical classification framework (summarized in Fig. 1) to detect trees from a time series of NAIP aerial imagery, addressing biases from sensor and environmental variabilities across survey years. The framework integrates four sequential steps. Step 1 is to preprocess data, including calculating spectral indices for classifications. Step 2 and Step 3 involve two-stage classifications respectively: ***Stage I****-* delineating vegetated areas through adaptive thresholding and noise reduction (e.g., shadow removal, sensor saturation correction); ***Stage II****-* differentiating trees from grassland using spectral-textural metrics. The final Step 4 is assessing, refining, and finalizing classification results. Thresholds used in ***Stage I*** and ***II*** are determined through a combination of automated initial estimations and manual adjustments. First, an initial threshold estimate is generated using unsupervised classification of randomly selected reference data, incorporating features such as NDWI, NDVI, brightness, and texture. However, the initial thresholds are highly dependent on the representativeness of samples. To ensure accurate identification of trees across the target domain, manual adjustment of the thresholds based on classification results is necessary. This hybrid approach balances time efficiency with the precision required to achieve optimal results.

Specifically, ***Stage I*** (Step2) focuses on broader vegetation delineation. Vegetated and non-vegetated surfaces were differentiated using the Normalized Difference Vegetation Index (NDVI) and Normalized Difference Water Index (NDWI) (Fig. 1, Steps 1–2, see NDVI and NDWI definitions in Table 1). Sensor-specific radiometric biases (e.g., spectral calibration differences) were minimized through the hybrid approach for each survey year. Additional inconsistencies from shadows, resolution shifts, and environmental noise were mitigated using morphological filtering and manual consistency checks. ***Stage II*** (Step 3) aims to differentiate tree canopies and grassland. Within vegetated areas, trees were distinguished from grassland using texture analysis and spectral brightness (Fig. 1, Step 3-4, see the index definitions in Table 1), leveraging principles from Myeong *et al*.^48^ and Qian *et al*.^49^. High-resolution imagery reveals that grassland exhibits smoother textures and higher brightness due to uniform light reflectance, while tree crowns appear darker and rougher because of vertical leaf occlusion and shadowing within the canopy. Annual thresholds for texture and brightness were similarly calibrated using the hybrid approach to account for interannual variability in imagery conditions. Note that despite the thresholds were determined independently for each year. We have verified the invariant reference features (e.g., permanent water bodies, well-established urban areas) as anchors to ensure consistency of classification results. Validation included iterative sensitivity testing and cross-referencing with ground-truth data.

**Table 1:** Description of selected indices for classifications.

Index	Description	Characteristics		Method
		Strengths	Limitations	
Normalized Difference Vegetation Index (NDVI)^50^	Estimates vegetation greenness, density, and health by quantifying chlorophyll activity.	+Distinguishes vegetated (positive values) vs. non-vegetated surfaces (negative/zero values). +Widely validated for canopy health assessment.	-Saturation in dense canopies. -Ambiguity in low/high biomass regions.	(NIR−Red)/(NIR + Red)
Normalized Difference Water Index (NDWI)^51^	Identifies open water features by enhancing contrast between water and dry land/vegetation.	+Highlights water bodies (positive values). +Effective in urban hydrologic mapping.	- Sensitive to built-up surfaces, risking false water detection.	(Green−NIR)/(Green + NIR)
Texture^52,53^	Quantifies spatial heterogeneity in canopy structure using grayscale variability. Tree crowns exhibit roughness due to vertical leaf occlusion.	+Differentiates trees (rough texture) from grassland (smooth texture). +Robust to spectral variability.	-Computationally intensive. - Requires high-resolution imagery.	Second-order co-occurrence metrics (NIR band) considering 8-pixel adjacency kernel.
Brightness	Amplifies spectral differences between vegetation types. Grassland exhibits higher brightness due to uniform structure and their spectral characteristics.	+Enhances contrast between trees (low brightness) and grassland (high brightness). +Simple to compute.	- Sensitive to shadows and solar angle variations.	∑(Red^2^ + Green^2^ + Blue^2^ + NIR^2^)

The final maps classify land cover into three categories: ***tree canopies, non-vegetation area, and grassland***. ***The tree canopies*** represent pixels dominated by dense, overlapping vegetation crowns characteristic of woody plants. ***The non-vegetation*** category encompasses pixels covered by impervious surfaces (e.g., buildings, roads), water bodies, bare soil, or other non-vegetated features. ***The grassland*** category includes remaining pixels, primarily low-lying herbaceous vegetation, encompassing both natural grassland and artificially cultivated grassland. This tripartite classification balances specificity and practicality for urban forestry management and environmental analysis.

#### Step 1 preprocessing data

This step involves calculating four key metrics—NDVI, NDWI, texture, and brightness—from NAIP multispectral imagery for each study year (see Table 1). NDVI, a widely validated indicator of vegetation density and vigor^50^, is specifically designed to measure vegetation abundance and indirectly help distinguish non-vegetation. NDWI effectively reduces misclassification errors by differentiating water bodies in hydrologically complex urban areas like Orlando^51^. Texture, derived from second-order co-occurrence matrices applied to the NIR band, quantifies spatial heterogeneity in canopy structure using an 8-pixel adjacency kernel^52,53^. The indicator of brightness is defined for this study as the sum of squared digital values across all four NAIP spectral bands. This brightness metric is used to amplify spectral contrasts between tree crowns (lower brightness due to shadowing, and higher leaf density with stronger radiation absorption, etc.) and grassland (higher reflectance uniformity, pigment concentration differences, etc.).

#### Step 2 & 3 classification

Thresholding was performed separately for each survey year to mitigate sensor-induced biases and other environmental condition changes across the Orlando metropolitan area. Thresholds for vegetation/non-vegetation (***Stage I***) and tree/grassland (***Stage II***) classifications were iteratively optimized using the hybrid approach, informed by automated initial estimation, visual interpretation and stratified random sampling from ground truth. ***Stage I*** determines thresholds to the NDVI and NDWI values with a simple binary decision tree classification to separate vegetation from non-vegetation, followed by noise removal and consistency adjustment. Validation of at least 2,000,000 samples achieved > 97% overall accuracy in distinguishing vegetated from non-vegetated surfaces. For ***Stage II***, two decision nodes were designed, utilizing ~200,000 samples, yielding 78–89% accuracy in differentiating trees from non-tree across the study period (2013–2021). *Node 1* refines the initial non-vegetation mask from ***Stage I*** by combining the existing mask with a brightness threshold. This is because some building materials might not have the same spectral curve as vegetation, but the ratio of NIR to red can sometimes be enough to trigger a higher-than-expected NDVI^54^. However, those building materials might differ significantly in their overall brightness. *Node 2* applies thresholds to the brightness and texture values within the refined vegetation mask to separate tree canopies. Thresholds were adjusted to account for interannual variations in sensor specifications, solar geometry, and phenology, with final selections by visual comparison with high resolution reference imagery and cross-validating against validation samples. Detailed thresholds, validation metrics, and sensitivity analyses are provided in Table 2 and the validation subsection.

**Table 2:** Thresholds identified to delineate vegetation from non-vegetation (Veg. versus Non-Veg.) in ***Stage I*** and to distinguish tree canopies and grassland in ***stage II***.

Year	Spatial Res.	Stage I: Veg. vs. Non-Veg.		Stage II: Tree-Grassland Separation		
				Non-Vegetation	Tree Canopies	Grassland
2013	1 m	NDWI > 0 and NDVI < −0.05	Noise Removing & Consistency checking	Brightness > 150,000 **or** Non-Veg = 1	Brightness < 50,000 **or** Texture > 50	Remaining pixels not classified above
2015		NDWI > −0.05 and NDVI < 0.05		Brightness > 150,000 **or** Non-Veg = 1	Brightness < 70,000) **or** Texture > 50	
2017		NDWI > −0.1 and NDVI < 0.15		Brightness > 120,000 **or** Non-Veg = 1	Brightness < 80,000 **or** Texture > 50	
2019	0.6 m	NDWI > −0.05 and NDVI < 0.05		Brightness > 100,000 **or** Non-Veg = 1	Brightness < 55,000) **or** Texture > 50	
2021		NDWI > 0 and NDVI < 0		Brightness > 120,000 **or** Non-Veg = 1	Brightness < 70,000) **or** Texture > 50	

**Fig. 2:**
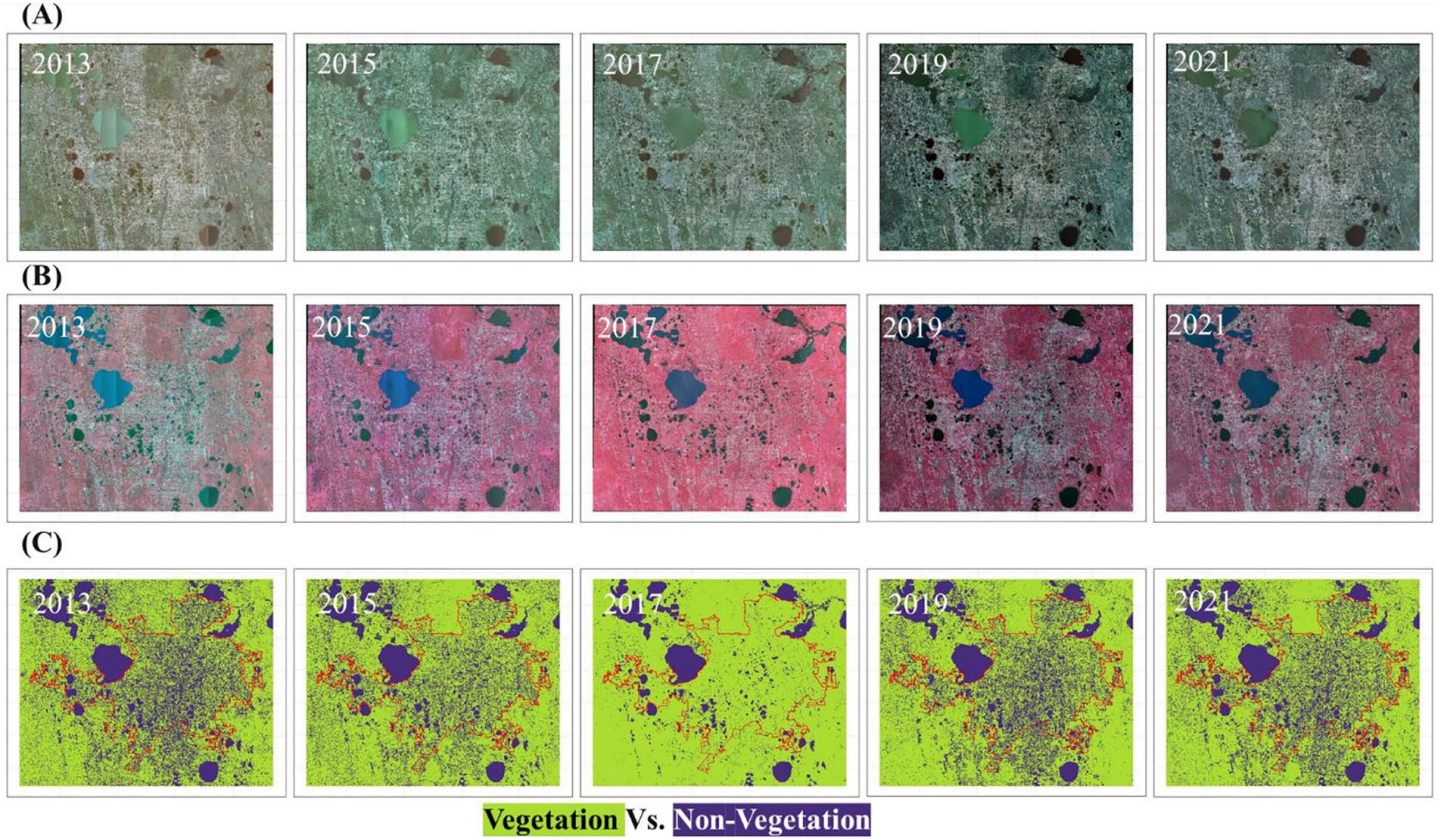
Comparison of input imagery and Stage I classification outputs (2013–2021). (**A**) True-color composites (RGB) of NAIP aerial imagery for each survey year. (**B**) False-color composites (NIR-R-G) highlighting vegetation vigor (red hues) and urban features (cyan/blue). (**C**) Initial Stage I classifications (vegetation vs. non-vegetation) before post-classification correction, illustrating sensor saturation artifacts in 2017 for overestimation of vegetations.

The Step 2 (***Stage I*** classification) NDVI and NDWI thresholds (Table 2) achieved multi-temporal consistency in distinguishing vegetated from non-vegetated pixels across most years, except for 2017 (Fig. 2). The 2017 NAIP imagery exhibits sensor saturation in the near-infrared (NIR) band, where the sensor’s radiometric response plateaued, failing to resolve subtle reflectance differences between dense vegetation and urban built-up surfaces. This saturation causes anomalously high NIR reflectance in impervious areas (e.g., rooftops, roads), leading to overestimated vegetation cover that obscured urban land cover distinctions (Fig. 2, 2017 panels). To mitigate this, a post-classification correction workflow was implemented, combining spatial noise removal and consistency checks. This adjustment ensured alignment with the classifications of historical and subsequent years, preserving dataset integrity across the time series.

**Fig. 3:**
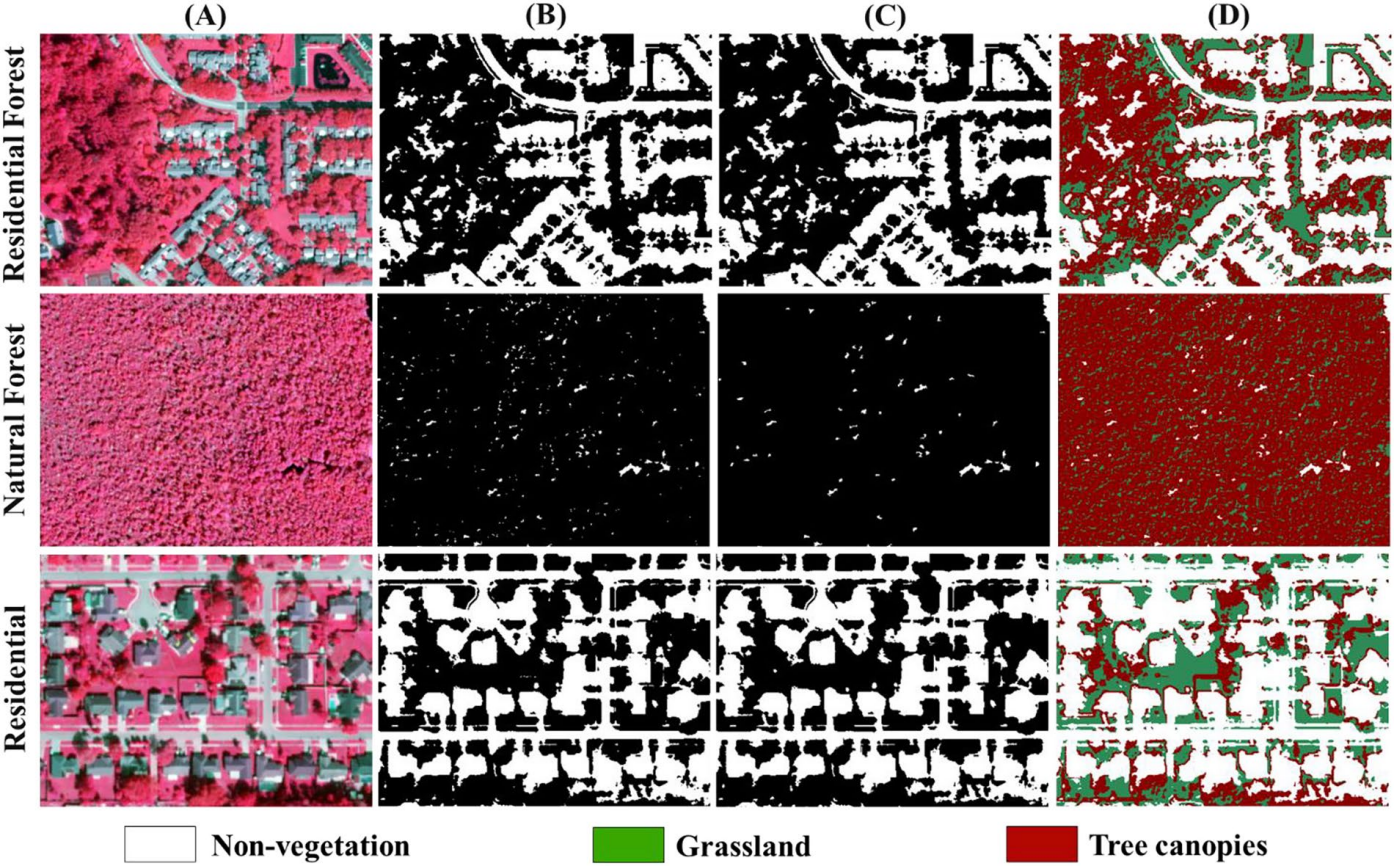
Noise reduction effects in three representative Orlando landscapes (Year 2021). Column (**A**) False-color composite (NIR-R-G) highlighting vegetation. (**B**) Initial Stage I binary decision tree classification (vegetation vs. non-vegetation) before noise removal, showing fragmented misclassifications from shadows and impervious surfaces. (**C**) Refined vegetation delineation after adaptive sieve filtering, resolving isolated non-vegetated pixels (e.g., tree occlusion shadows). (**D**) Final classification integrating ***Stage I*** and ***Stage II*** (tree/grassland) outputs, demonstrating improved urban canopy continuity.

To reduce classification artifacts from high-resolution imagery (e.g., shadow occlusion, fragmented land cover), we applied morphological sieve filtering with adaptive spatial kernels. This process merges isolated pixel groups smaller than a defined threshold into adjacent dominant classes. The kernel size in pixels for the morphological filtering remains constant at 3 × 3. This is supported by extensive research and practical applications that have demonstrated the effectiveness of 3 × 3 kernels in foundational image processing techniques, such as edge detection^55,56^ and noise reduction^57^. It represents a good balance between capturing local spatial information, maintaining computational efficiency, and enabling effective noise removal. In our specific application, a 3 × 3 kernel effectively removed small, isolated noise features while preserving the structural integrity of larger tree canopy areas. Larger kernels tended to over-smooth the data, while smaller kernels were less effective at noise removal. It is noted that the spatial extent of the kernel, measured in meters, changes across different years (2013–2021) due to the variation in image resolutions. Given NAIP’s resolution shift from 1 m (pre-2018) to 0.6 m (post-2018), kernel sizes were scaled accordingly: 3 × 3 m^2^ (3 × 3 pixels at 1 m) and 1.8 × 1.8 m^2^ (3 × 3 pixels at 0.6 m). Non-vegetated pixel clusters smaller than 9 contiguous pixels (<9 m^2^ pre-2018; <3.24 m^2^ post-2018) were reclassified as vegetated to mitigate false positives from tree occlusion shadows and sensor noise (Fig. 3). The sieve filter addressed isolated non-vegetated pixels by accounting for resolution differences, particularly resolving issues caused by shadow occlusion from tree canopies in relatively well vegetated areas.

**Fig. 4:**
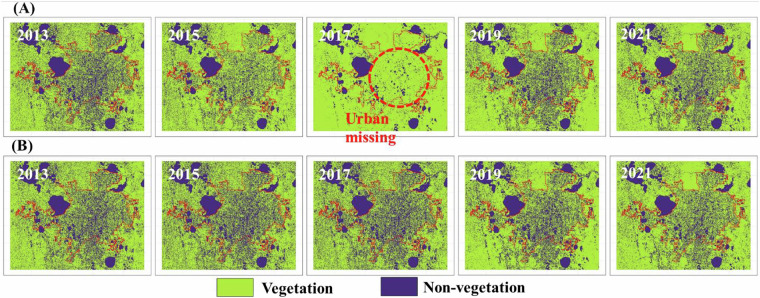
Consistency adjustment effects in Orlando landscapes. (**A**) post-noise removal highlighting the inconsistency between the ***Stage I*** non-vegetation mask in 2017 and the ***Stage I*** non-vegetation masks in other years. (**B**) Post-consistency adjustment resolves the “data saturation” problem and improves the consistency of 2017 vegetation mask.

The 2017 data exhibited a unique “data saturation” issue, resulting in the ***Stage I*** non-vegetation mask with significantly lower overlap compared to other years. Specifically, the 2017 mask showed only 34.97%, 43.57%, 39.01%, and 41.6% overlap with the 2013, 2015, 2019, and 2021 masks, respectively. In contrast, the overlap between non-vegetation masks from any two of those other four years consistently exceeded 65%. To address this 2017-specific saturation problem (Fig. 2), we implemented a multi-mask correction approach. This involved integrating four masks: (1) the 2013 ***Stage I*** non-vegetation mask (*M*_*2013*_), (2) the 2015 ***Stage I*** non-vegetation mask (*M*_*2015*_), (3) the 2017 ***Stage I*** non-vegetation mask (*M*_*2017*_), and (4) a 2017 low NDVI mask (*M*_*NDVI*_) where NDVI < 0.15. The historical and current non-vegetation masks, representing stable features like permanent water bodies and urban areas, served as “anchors” to ensure consistency in the 2017 mask (see red circle in Fig. 4, 2017). To minimize temporal bias, these non-vegetation masks were applied only to regions with low NDVI values, as identified by the 2017 low NDVI mask. The NDVI threshold of 0.15 was determined from the 2017 NDVI histogram of known vegetated samples, effectively identifying non-vegetated areas. The substantial improvement in urban area representation in Fig. 4(A) after correction, coupled with a high overall accuracy of 98.4% for vegetation/non-vegetation in Table 4, demonstrates the efficacy of our saturation correction. The detailed algorithmic process is represented by the following mathematical formula:

$${M}_{{corrected}}={M}_{{NDVI}}{\rm{AND}}\,\left({M}_{2013}{\rm{OR}}\,{M}_{2015}{\rm{OR}}\,{M}_{2017}\right)$$

**Fig. 5:**
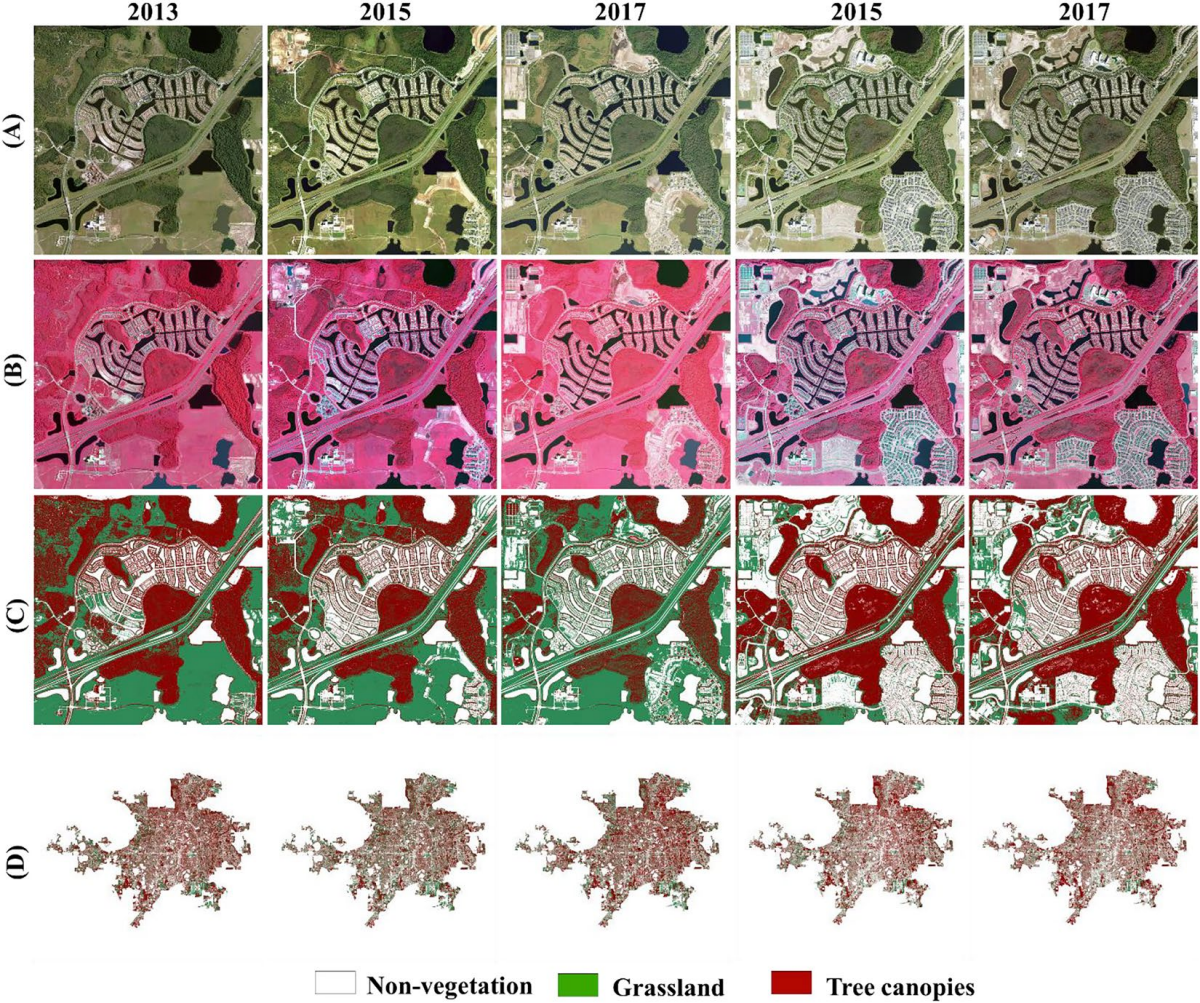
Example of tree canopy dynamics over one representative scene in the Orlando metropolitan area. (**A**) True-color composites (RGB) and (**B**) False-color composites show each year’s conditions, and there is a clear calibration inconsistency present among five surveyed years. (**C**) Final results show tree canopies, grassland, and non-vegetated areas changes over time. (**D**) Final results show tree canopies, grassland, and non-vegetated areas changes over the entire metropolitan area.

High-resolution aerial imagery reveals distinct spectral and textural contrasts between trees and grassland, as demonstrated by variations in brightness (spectral reflectance) and canopy roughness^48,49^. These metrics were integrated into a decision tree classifier for ***Stage II***, which categorized vegetated pixels into tree canopy or grassland using threshold-based criteria (Table 2). Brightness thresholds exploit the higher reflectance of grassland in visible/NIR bands, while texture metrics capture the structural heterogeneity of tree crowns. This biennial classification framework enables precise tracking of canopy dynamics across space and time. As a result, the final datasets capture the tree canopy dynamics across the Orlando metropolitan area that are summarized in Table 3. Figure 5 shows a detailed example of geolocated change trajectories of tree canopies, and the final results of the entire domain.

**Table 3:** Statistics of tree canopy dynamics over Orlando metropolitan area and the City of Orlando.

Domain	2013	2015	2017	2019	2021
Orlando Metropolitan Area	41.1%	34.6%	35.9%	36.4%	38.2%
Orlando (city)	39.5%	35.2%	35.9%	35.9%	37.8%

## Data Records

The dataset is hosted in the public data repository–Zenodo^58^. Table 4 summarizes the data size, format, and class information.

**Table 4:** Dataset Description.

File Name	Description	File Size	Properties		
2013	Biennial tree canopy maps from 2013 to 2021 of the Orlando metro with a spatial resolution of 1 m or 0.6 m. All the datasets are in ENVI format (.img + .hdr)	5.65 GB	Value	Category	Description
2015			3	Tree canopies	Tree canopies represent pixels dominated by dense, overlapping vegetation crowns characteristic of woody plants
2017			2	Non-vegetation Grassland	Non-vegetation encompasses pixels covered by impervious surfaces (e.g., buildings, roads), water bodies, bare soil, or other non-vegetated features
2019		15.6 GB			
			1	Grassland	Grassland is primarily composed of low-lying herbaceous vegetation, encompassing both natural grasses and artificially cultivated grasses
2021		15.7 GB			

## Technical Validation

Two validation strategies were used to demonstrate the quality of the dataset. First, we directly validated the detection rates based on collected ground truth using sample representative areas over the large domain. we also compared our product estimates from other existing products.

### Direct validation using ground truth data

**Fig. 6:**
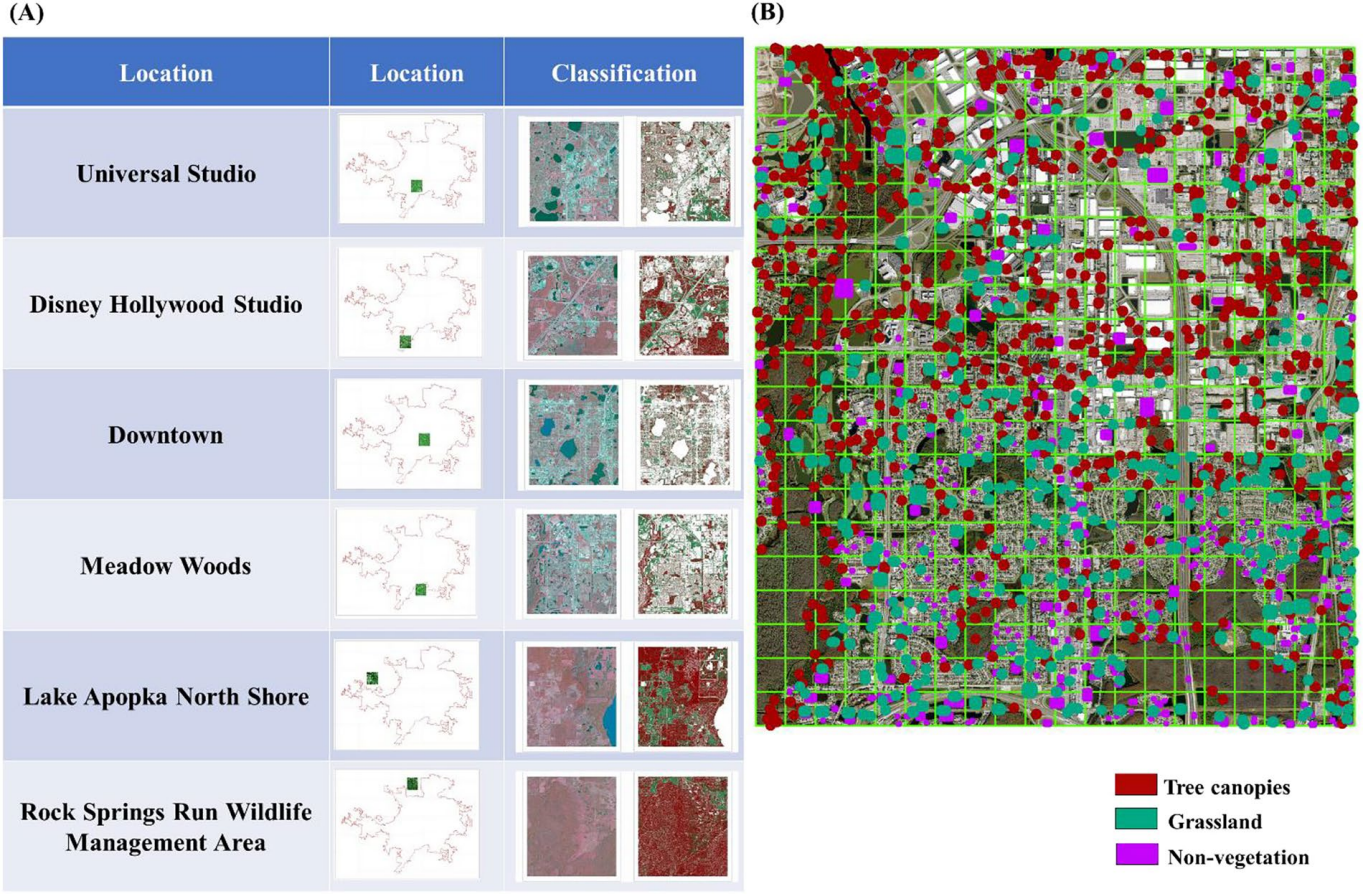
Validation sampling design and examples. (**A**) Spatial distribution of six representative tiles across the Orlando metropolitan area, selected to capture diverse landscapes. Classification results for each tile illustrate accuracy assessment coverage. (**B**) Validation sample distribution within a single tile. Samples were visually inspected and stratified across land cover classes (tree canopies, non-vegetation and grassland) to ensure spatial and thematic representativeness.

We manually collected ~3,600 polygons across static land area (unchanged from 2013–2021) to validate classification accuracy. In validation processing, pixel-level ground truth samples were used, which are involved in two stages validation, are listed in Table 4, including millions of pixels for ***Stage I*** validation and hundreds of thousands of pixels for ***Stage II*** validation. To ensure geographic and land-use representativeness, six tiles spanning distinct landscapes in the Orlando metropolitan area—urban core, suburban, rural, and recreational zones (e.g., theme parks)—were selected (Fig. 6). Each tile was subdivided into a 20 × 20 grid (400 cells), with validation samples visually inspected and systematically selected within each cell. This stratified sampling framework ensures spatially balanced representation of land cover heterogeneity, enhancing reliability over purely random sampling approaches.

We established ground truth through visual interpretation of the original NAIP imagery for every other year during 2013–2021. The accuracy assessment for each year is independent, directly comparing the classified results to the actual ground truth. While these ground truth samples are static, they do not lead to accuracy overestimation in our case, as we did not employ these samples for training and classifications (e.g., supervised machine learning approaches). Accuracy overestimation from static samples typically occurs when models memorize specific patterns in these machine learning approaches, resulting in overfitting and poor generalization on unseen data. Our methodology diverges significantly. Classification results are determined by distinct, year-specific thresholds, meticulously adjusted to account for inherent inconsistencies in the imagery’s spectral and radiometric responses. This year-by-year threshold adjustment ensures that the classification process is entirely independent for each time period and, importantly, independent from the validation samples themselves, effectively mitigating the risk of accuracy overestimation.

Besides, a significant advantage of using these temporally consistent ground truth samples is that it allowed us to rigorously evaluate the consistency of our classification methodology. Our primary focus was to ensure that trees were consistently and accurately categorized across different years (2013–2021) and image resolutions (1 m to 0.6 m). By verifying those unchanged ground truths were consistently classified, we strengthened the reliability and robustness of our classification approach across varying data conditions. This allows us to evaluate classification accuracy while accurately accommodating potential real-world spectral changes that may have occurred over time. Confusion matrix is generated to show the true positives, false negatives, false positives, and true negatives by comparing the final classification results and the collected ground truth samples. The summarized performances in overall accuracy are shown in Table 5.

**Table 5:** Overall accuracy of vegetation and tree canopy detections.

Overall Accuracy	Vegetation	Tree Canopies	Sample Size (pixels)
2013	99.5%	78.3%	Vegetation delineation: 2,073,765 Tree canopy detection: 176,769
2015	99.1%	82.3%	
2017	98.4%	83.7%	
2019	97.2%	87.8%	Vegetation delineation: 5,620,052 Tree canopy detection: 477,015
2021	98.1%	88.8%	Vegetation delineation: 5,760,702 Tree canopy detection:491,705

### Comparisons with other data sources

To establish temporal context, we compared our findings with the Orlando City data from 2018^59^. The Orlando City 2018 Community Action Plan reported an increase in tree coverage to 32% by 2018, compared to 2012 of 23%, attributed to the city’s tree planting programs, using the USDA’s i-Tree tool. In contrast, our results indicate a higher tree coverage of approximately 36% in both 2017 and 2019. Furthermore, our data captures the nuanced dynamics of tree canopy change, revealing losses due to rapid urban development between 2013 and 2015, followed by restoration efforts in subsequent years. These detailed temporal patterns highlight the uneven distribution of canopy changes across the city. Supporting our findings, aggregated trends from Global Forest Watch (2000–2020) show a marginal net gain in metropolitan tree cover, aligning with our estimations and reinforcing the validity of our results.

**Fig. 7:**
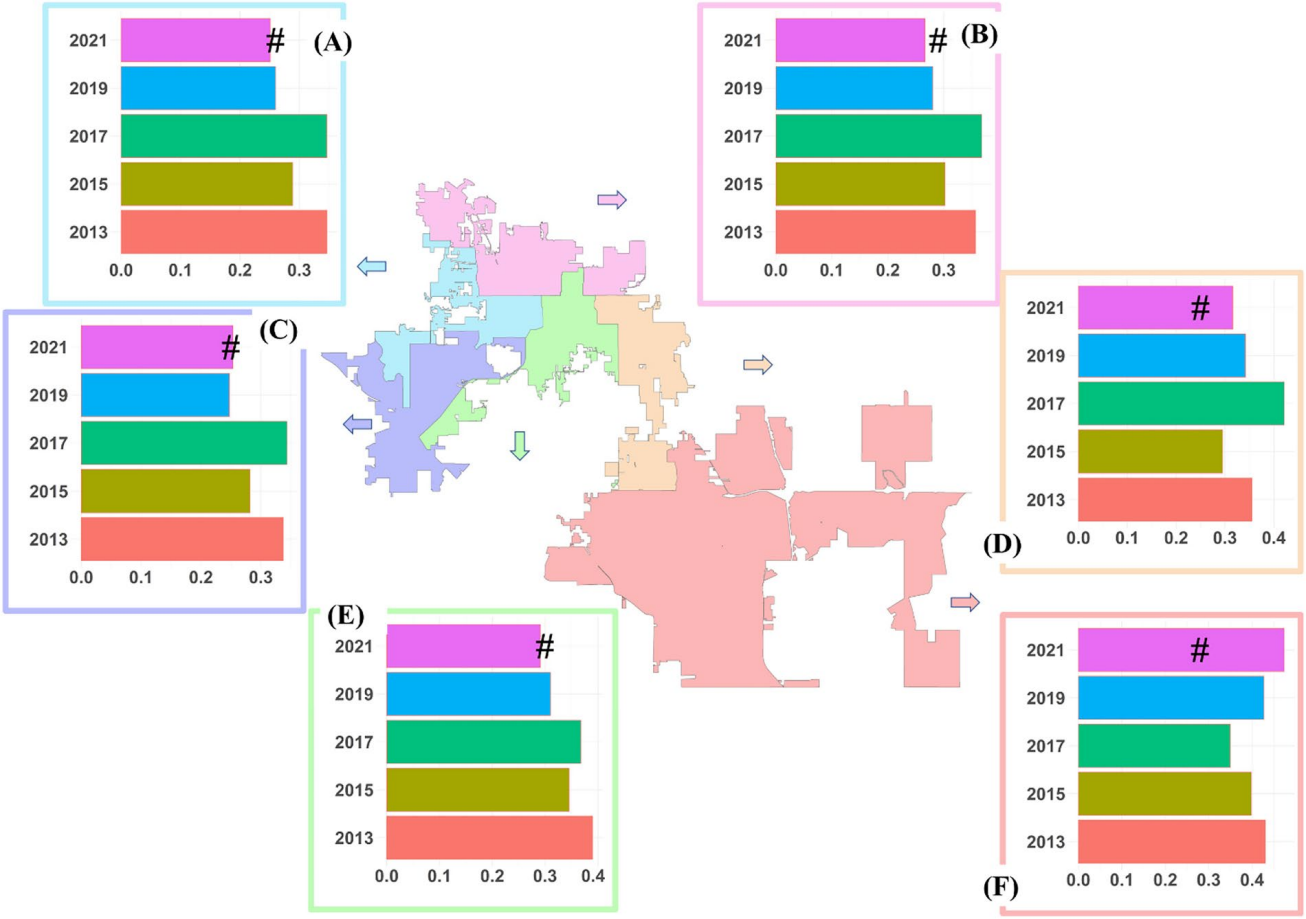
Statistics of tree coverage ratio over six commissioner districts in Orlando City from our datasets and comparison of results from 2021 urban tree coverage from PlanIT Geo (shown as # symbol).

Spatially, we summarize the tree coverage ratios across six commissioner districts in Fig. 7. Comparing our estimates with 2021 estimates from PlanIT Geo^60^ (based on aerial imagery, only available in 2021) (Fig. 7, A-F #, vs. colored bar plot of our results), overall coverages closely match in highly urbanized districts (district A-E in Fig. 7). However, a much higher tree coverage is found over less developed southeastern Orlando (see Fig. 7, district F). Partly, we have a different classification scheme. PlanIT Geo distinguishes shrubs from trees but we lump two types together. The discrepancy thus becomes larger in areas of high vegetation complexity due to such difference in classification schemes. Note that, although spatiotemporally our estimations of tree coverage show a good match with the above-mentioned products, but these existing products derived from high-resolution imagery are only available at the aggregated scale, so our comparison is performed at the relevant scales.

Specifically, the 2021 PlanIT Geo report was the only available version and dataset that utilized NAIP imagery for sub-meter tree classification over Orlando; no other comparable sub-meter products were publicly accessible. As PlanIT Geo is a commercial entity, we believe our product contributes valuable, publicly accessible data for those requiring detailed tree coverage information for comparison and analysis. We remain committed to conducting a more robust quantitative comparison when additional suitable data becomes available.

### Further discussions and limitations

Our biennial sub-meter tree coverage dataset offers the spatiotemporal detailed information of tree canopies. Using this high spatiotemporal resolution product should notice that:

1. While the transition from 1 m to 0.6 m resolution NAIP imagery yields negligible improvement for broad vegetation/non-vegetation classification, which already achieves approximately 99% accuracy, it significantly enhances feature extraction for tree and grassland classification. Simulated results demonstrate an accuracy increase from approximately 80% to 90%. This enhanced spatial detail enables our algorithms to derive more precise and informative features, leading to improved classification performance in 2019 and 2021. That is, although the 1-meter resolution is sufficient for accurately resolving individual trees, minor differences in tree canopy detection are expected in 0.6-meter resolution. Therefore, our final products, validated using data from both resolutions and exhibiting comparable accuracy as presented in Table 5, are designed for tract or neighborhood-level applications, rather than individual tree canopy analysis.
2. Orlando domain experienced a rapid development during the surveyed periods. We observed a vegetation clear for construction and development, like building structures and infrastructure. And then, once the construction is complete, trees are planted in the newly developed area to restore greenery and mitigate the environmental impact of the development. In most of the cases, the restoration cannot reach to their original status due to limited room (see Fig. 8). Thus, interpreting spatiotemporal changes in tree coverage requires careful consideration of the underlying causes. For example, tree loss may indicate development, while tree gain may reflect post-construction replanting. Furthermore, while tree canopies are defined in this study as pixels dominated by dense, overlapping vegetation crowns of woody plants, including large shrubs, potential confusion between these categories could lead to canopy cover overestimation. Although LiDAR data could enhance tree-shrub differentiation, its limited availability that matches the time series of optical data make it challenging to realize in this product. Future research could refine tree/shrub classification through alternative features like advanced texture analysis.

**Fig. 8:**
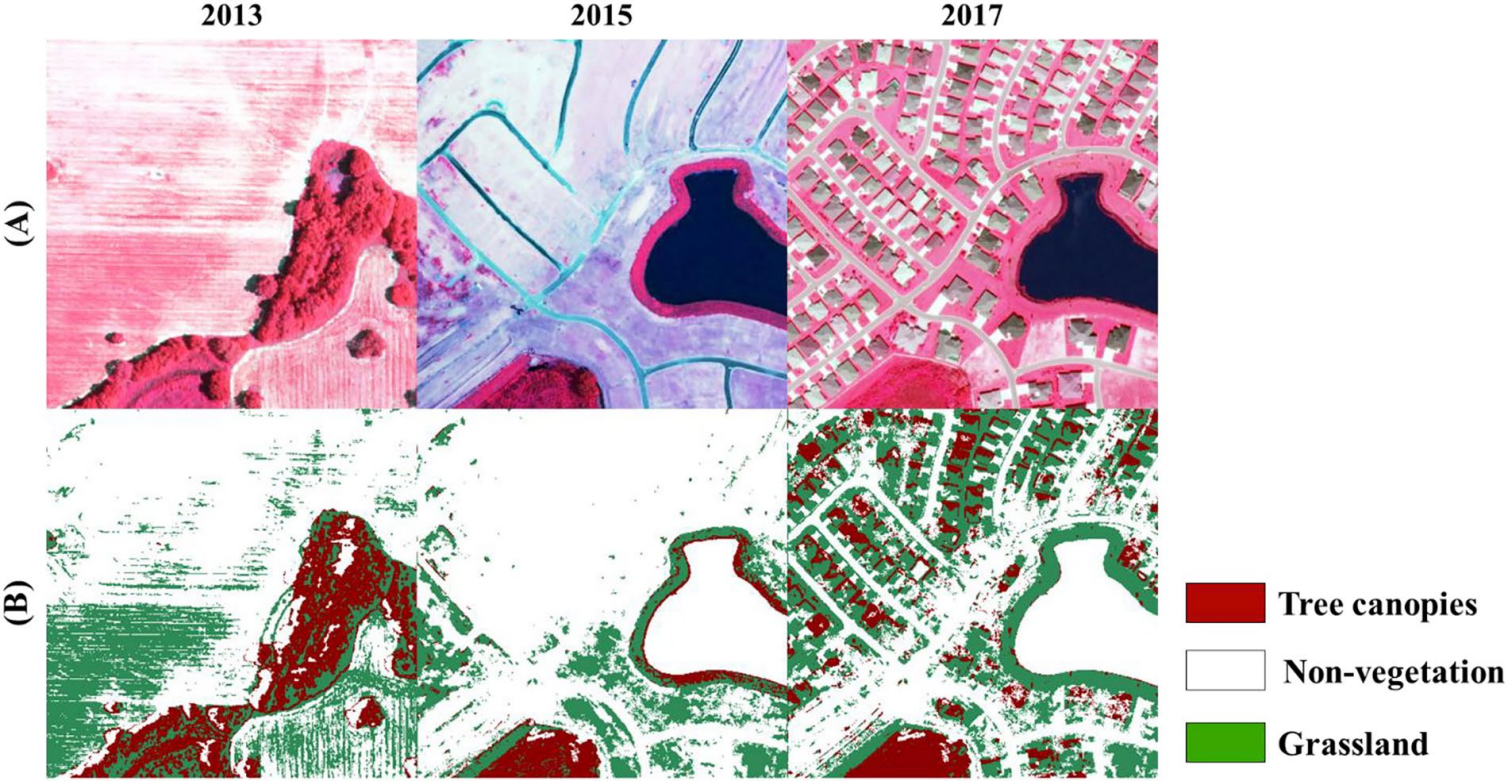
Land cover transitions in a developing urban site (2013–2017). (**A**) False-color composites show each year’s conditions. (**B**) Final results show tree canopies, grassland, and non-vegetated areas changes over time, illustrating tree canopy dynamics linked to construction activities. 2015 image shows initial tree loss during site preparation and 2017 image reveals canopy recovery post-construction, likely due to municipal replanting mandates or landscaping.
3. The high-resolution images depict sharper signals of more detailed trees as well as their shadow. Unlike many Asian cities featured with skyscrapers, most residential areas have one or two floor buildings that are similar or even lower than the tree canopies. The shadow castings on tree canopies are not common. The shadow mostly impacts the accuracy of the lower height objects (e.g., grassland and other pavement etc.) near tall trees, which is out of our primary objective to ensure the consistent and accurate categorization of trees across different years (2013–2021) and image resolutions (1 m to 0.6 m), rather than focusing on all land cover types. It also partially impacts the tree canopy coverages as well when trees have different heights or relatively close to each other when shadow casts on certain directions. These uncertainties can cause some inconsistency due to changes of shadow trajectory in different years. Figure 9 A and B show example due to different solar illumination angles in multi-temporal classification comparison. This shadow change issue is more easily distinguished and modified at large scale forest, where the leaves of trees have more mutual occlusion in vertical space. This is because condense mutual occlusion in vertical space causes smaller size shadow noises, which could be removed by our Sieve filter noise removing process (Fig. 3). However, large shadows (greater than 3*3 pixels) from highly fragmented greenspace patches (e.g., isolated tall trees or buildings), contrarily, alter the appearance of features and obscure details, leading to misclassification for lower height land cover types.

**Fig. 9:**
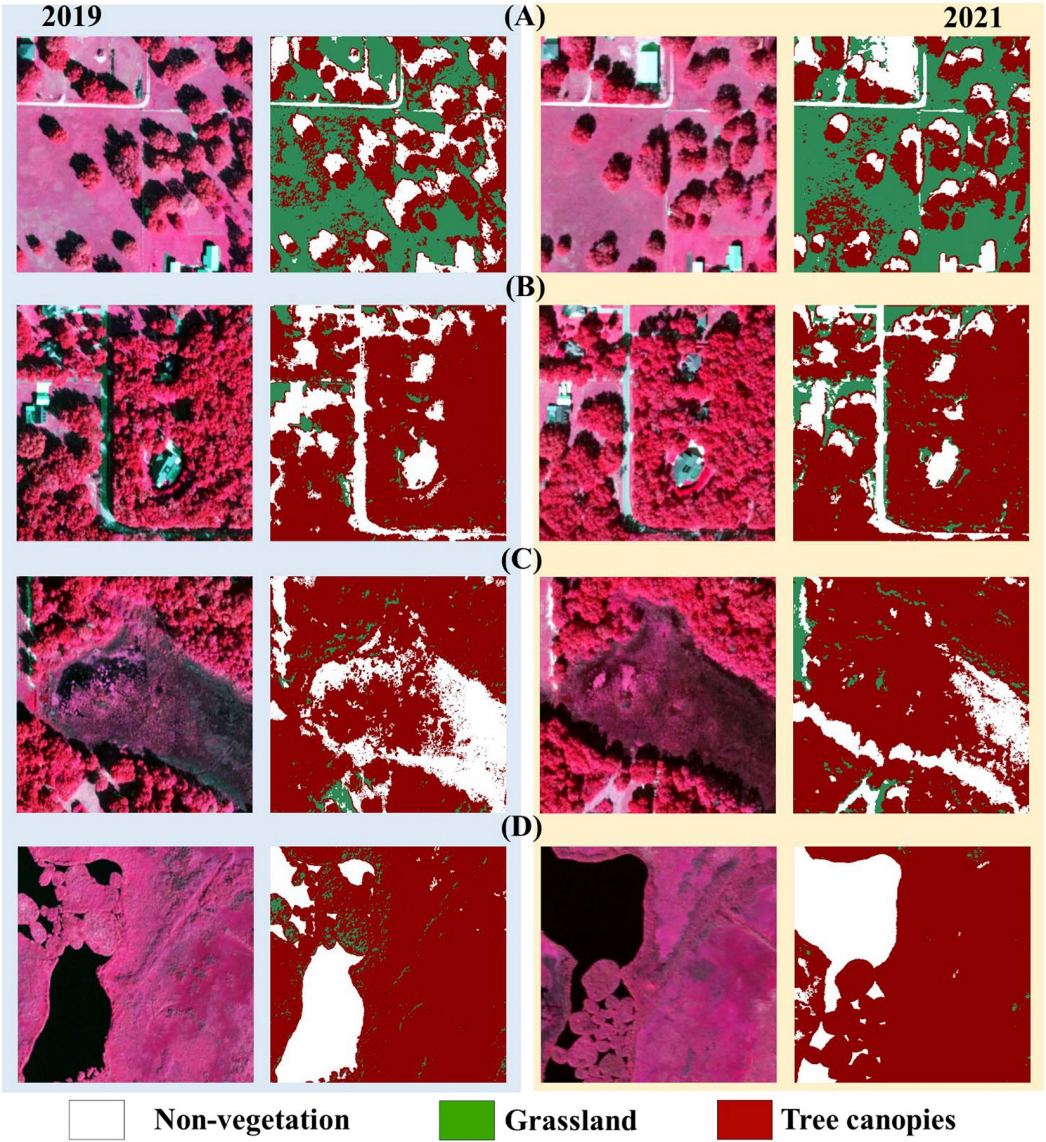
Examples of possible temporal variations in land cover with using this high spatiotemporal resolution product. (**A**) Shadow in different directions (solar azimuthal angle); (**B**) Shadow in different sizes (solar zenith angle); Vegetation differences caused by different water conditions (**C**) and distributions (**D**).
4. This study did not explicitly account for seasonal changes due to the acquisition strategy of NAIP imagery. NAIP captures aerial images during the peak growing season, typically every other year, across the continental United States, which is designed to reduce within-dataset seasonal variability. Furthermore, temporally consistent ground truth samples were used for validation, ensuring a robust assessment of our classification methodology’s consistency. However, it’s crucial to acknowledge that NAIP imagery for Orlando is collected during the warm and wet season (May to November). This period coincides with significant hydrological fluctuations in wetlands, such as marshes, swamps, and bogs, which are highly sensitive and dynamic ecosystems. These changes impact vegetation health and radiometric consistency between multi-temporal images (Fig. 9 CD). While the NAIP acquisition strategy aims to minimize seasonal variability, the inherent dynamics of Orlando’s wetlands during the acquisition window introduce a degree of temporal heterogeneity that should be considered.

In general, our product achieves accuracy comparable to those reported in studies utilizing NAIP imagery and advanced methodologies like machine learning and deep learning^61,62^. Our product adopts a simple decision tree approach that provides a robust and efficient solution. For pixel-level classification of very high-resolution data, particularly for applications at the neighborhood level, the traditional decision tree approach, with its inherent interpretability and simplicity, is proved well-suited for such straightforward task. Furthermore, it offers the advantage of replicability for future tree cover mapping with minimal technical barriers for city applications. While advanced machine learning techniques offer superior transferability and generalization due to their capacity to learn robust feature representations and adapt to new domains^63^, their reliance on extensive training data posed a challenge in our context, which focused solely on tree canopy extraction. Implementing such methods would necessitate significant effort in preparing diverse and representative training samples, given the complexity of urban land cover.

## Usage Notes

The dataset is compatible with standard geospatial analysis tools, including scripting languages (e.g., R, Python, and MATLAB) and Geospatial software (e.g., ENVI, Arc Pro, and QGIS) for custom statistical analysis and visualization.

## Data Availability

The IDL (Interactive Data Language) code used to generate the tree canopy maps is publicly available on GitHub^64^ to ensure reproducibility. The repository includes two core scripts: Extract_index.pro and Hierarchical_classification.pro. The first script processes raw NAIP imagery into four derived indices—NDVI, NDWI, texture, and brightness. The second script implements the hierarchical classification framework (Hierarchical_classification.pro).
